# Predictive value of a severity-of-illness score for toxic epidermal necrolysis (SCORTEN) factors for in-hospital mortality in Stevens–Johnson syndrome/toxic epidermal necrolysis

**DOI:** 10.3389/fmed.2025.1735242

**Published:** 2026-01-12

**Authors:** Ekaterina Nikitina, Alexander Dushkin, Yuri Streltsov, Sergey Andreev, Tatiana Kruglova, Ulyana Markina, Marina Lebedkina, Alexander Fedorovsky, Alexander Karaulov, Maryana Lysenko, Daria Fomina

**Affiliations:** 1 Moscow Center of Allergy and Immunology, Moscow Clinical Science and Research Center 52, Moscow, Russia; 2 Department of Clinical Immunology and Allergology, The First Sechenov Moscow State Medical University (Sechenov University), Moscow, Russia; 3 Department of General Medicine No. 1, Moscow Clinical Science and Research Center 52, Moscow, Russia; 4 Intensive Care Unit No. 9, Moscow Clinical Science and Research Center 52, Moscow, Russia; 5 Department of Clinical Pharmacology, Moscow Clinical Science and Research Center 52, Moscow, Russia; 6 Project Office, Moscow Clinical Science and Research Center 52, Moscow, Russia; 7 Faculty of Medicine, Medical Research and Educational Institute, Lomonosov Moscow State University, Moscow, Russia; 8 Life Improvement by Future Technologies (LIFT) Center, Moscow, Russia; 9 General Therapy Department, The Russian National Research Medical University Named After N.I. Pirogov, Moscow, Russia; 10 Department of Pulmonology, Astana Medical University, Astana, Kazakhstan

**Keywords:** severity-of-illness score for toxic epidermal necrolysis (SCORTEN), Stevens-Johnson syndrome and toxic epidermal necrolysis (SJS/TEN), in-hospital mortality (IHM), predictive value, clinical outcomes

## Abstract

**Introduction:**

Stevens–Johnson syndrome (SJS) and toxic epidermal necrolysis (TEN) are life-threatening mucocutaneous reactions with high mortality. The A severity-of-illness score for toxic epidermal necrolysis (SCORTEN) scale remains the principal tool for early estimation of in-hospital death risk. The study was aimed to assess the predictive value of individual SCORTEN components for hospital mortality in a real-world Moscow cohort and to characterize demographic and etiologic factors associated with outcome.

**Methods:**

We retrospectively reviewed 150 adult patients (median age 50 years; IQR: 31–63; 59.3% female) admitted with SJS (45.3%) or TEN (54.7%) between 2019 and 2024. SCORTEN parameters were recorded within 24 h of admission. Univariate analyses (chi-square or Fisher's exact test) identified associations between each SCORTEN variable and in-hospital death. Kaplan–Meier survival curves and Cox proportional-hazards models quantified time-to-event outcomes and adjusted hazard ratios (HR).

**Results:**

Overall mortality was 18.7% (*n* = 28). In univariate analysis, age >40 years (OR: 3.53; *p* = 0.01), associated malignancy (OR: 3.35; *p* = 0.03), heart rate > 120 bpm (OR: 9.42; *p* < 0.001), serum urea >28 mg/dL (OR: 14.25; *p* < 0.001), and bicarbonate <20 mmol/L (OR: 7.25; *p* < 0.001) were significantly linked to death. In multivariate Cox regression, malignancy (HR: 3.57; *p* = 0.05), urea > 28 mg/dL (HR: 4.33; *p* = 0.03), and tachycardia (HR: 2.77; *p* = 0.04) remained independent predictors. Initial epidermal detachment, serum glucose, and age did not retain significance.

**Discussion:**

These findings support continued use of SCORTEN while highlighting the need to recalibrate or augment its parameters—particularly renal and oncologic variables—to improve risk stratification in current therapeutic contexts.

## Introduction

Stevens–Johnson syndrome (SJS) and toxic epidermal necrolysis (TEN), also known as Lyell's syndrome, are rare but severe delayed-type hypersensitivity reactions marked by high morbidity and mortality (1–3). The hallmark of both conditions is widespread epidermal and mucosal necrosis and detachment. SJS affects <10% of the body surface area (BSA), TEN involves >30%, and cases with 10%−30% BSA detachment are classified as SJS/TEN overlap (2, 4). Prompt diagnosis, immediate cessation of the causative agent, and early initiation of optimal therapeutic interventions are essential to mitigate the risk of systemic complications, including fluid and electrolyte imbalances, sepsis and septic shock, acute kidney injury, multiorgan failure (5). The cornerstone of SJS/TEN management is the immediate withdrawal of the suspected causative drug (6). The rarity and severity of SJS/TEN have precluded the conduct of large-scale randomized controlled clinical trials. Consequently, current treatment bases largely on retrospective case series and clinical experience (cyclosporine, systemic corticosteroids, intravenous immunoglobulins and TNF inhibitors) (6–14).

There remains an unmet need for a robust prognostic scale capable of accurately stratifying mortality risk in SJS and TEN. The SCORTEN is the current standard for assessing disease severity and estimating prognosis in these patients (15). The SCORTEN scores range from 0 to 7, with higher scores indicating more severe disease (16). Prognosis is influenced by various clinical and biological factors. In a multivariate analysis, SCORTEN has been demonstrated to be an effective tool for predicting outcomes in Taiwanese patients with TEN (17). However, other studies have indicated that SCORTEN may overestimate and underestimate actual mortality rates (18, 19). This is likely due to improvements in treatment algorithms since the SCORTEN was developed using data from patients admitted between 1979 and 1993 (16). Independent cohorts have demonstrated that the scale may in fact overestimate mortality in contemporary treatment environments and underestimate risk in specific subgroups, suggesting suboptimal calibration in real-world populations (19, 20). Systematic reviews and validation studies reported notable variability in its predictive accuracy, highlighting the influence of demographic factors, comorbidities, and advances in supportive care on SCORTEN performance (20–23). These findings underscore the need for continued reassessment and potential recalibration of existing prognostic tools to ensure accurate risk stratification in diverse patient populations. Subsequent attempts have been made to develop new prediction methods. One such method was developed by Noe et al. and is called ABCD-10 (19). This scale demonstrated comparable results to SCORTEN. However, studies indicated the higher prognostic accuracy of SCORTEN (21, 24).

The aim of this study was to apply the SCORTEN system, the best-known prognostic scale for SJS/TEN, to assess the predictive value of its seven-baseline clinical and laboratory criteria for in-hospital mortality risk prognostication during the acute phase of SJS/TEN.

## Materials and methods

### Patient population

A retrospective analysis of medical records from patients diagnosed with SJS or TEN was conducted using data from The Unified Medical Information and Analytical System of Moscow (EMIAS; EMИAC), covering the period from 2019 to 2024. The initial cohort included 267 patients aged 18 years and older who were hospitalized with primary diagnoses corresponding to ICD-10 codes L51.1 and L51.2.

**Figure 1:**
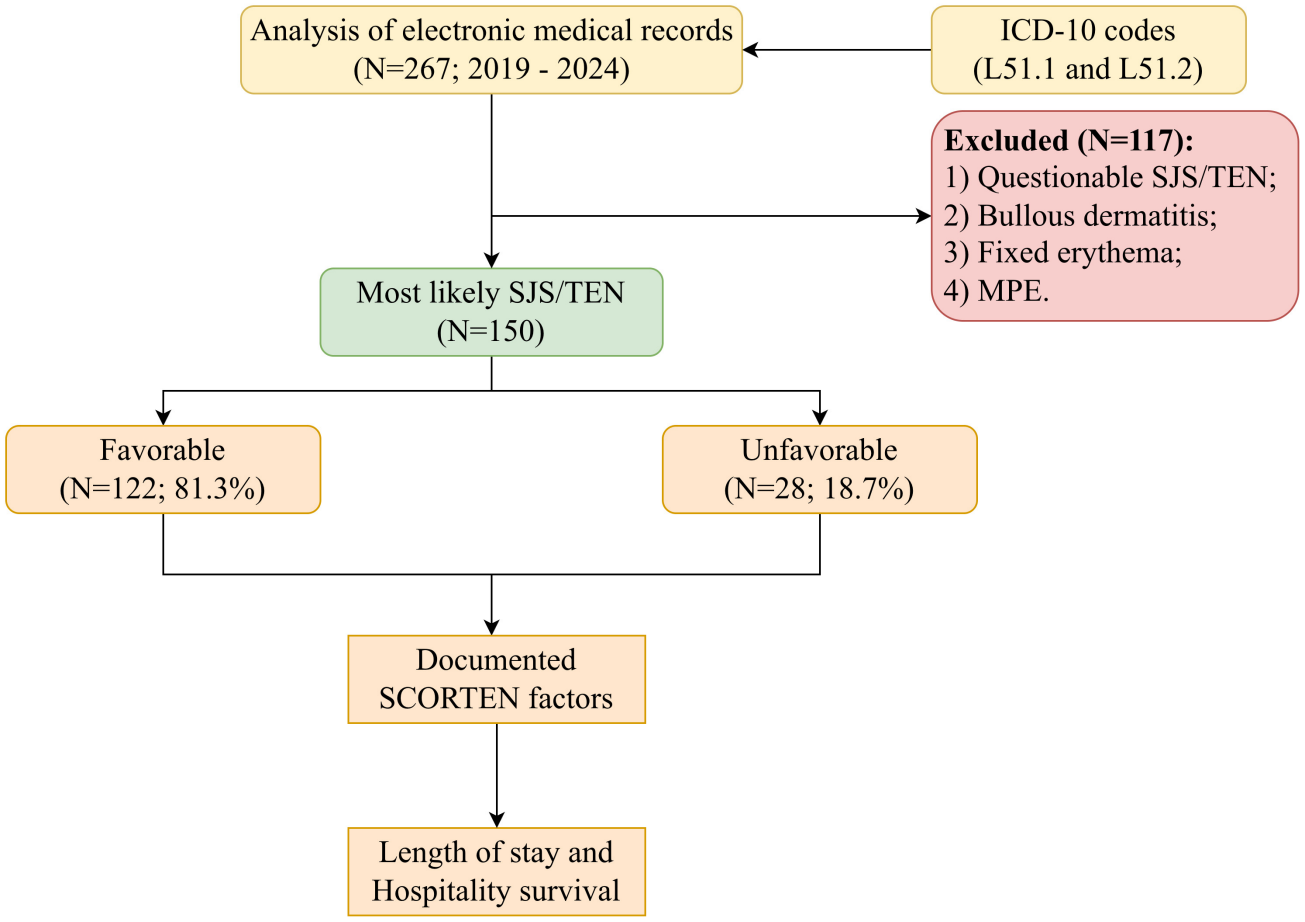
Flowchart illustrating the research methodology.

Due to the lack of universally accepted and standardized diagnostic criteria for erythema multiforme bullosa and toxic epidermal necrolysis, a detailed review of electronic medical records was performed. This evaluation included clinical and historical data such as disease duration, acute clinical presentation, pharmacological and infectious history, comorbidities, prior SJS/TEN diagnoses, and the patient's local status. The consistency between local status and SJS/TEN diagnostic characteristics was verified by multiple independent dermatology specialists, based on established clinical criteria including extent of epidermal detachment, mucosal involvement, and exclusion of alternative diagnoses such as erythema multiforme major. Histopathological confirmation was included when available. Patients with uncertain or questionable SJS/TEN diagnoses were excluded, and the number of excluded cases is reported in the participant flow diagram (Figure 1).

The final inclusion criteria required patients to be over 18 years of age, with well-documented clinical and historical data confirming consistency with SJS/TEN diagnostic characteristics. Additional requirements included acute disease onset, a reliable record of trigger exposure (including medication duration), and symptom onset within a defined latency period of 5–28 days following exposure. The latency period of 5–28 days and the definition of “acute onset” were selected based on established ALDEN criteria and epidemiological data on SJS/TEN. Most cases develop within 1–4 weeks of drug initiation, with shorter (<5 days) or longer (>28 days) intervals being atypical for first-time exposures. This definition ensures consistent case identification, minimizes inclusion of delayed or alternative etiologies, and supports reproducible application of causality assessment while acknowledging inherent selection constraints. In patients with a history of polypharmacy, the identification of the causative agent was determined using the Algorithm of Drug Causality in Epidermal Necrolysis (ALDEN) scoring system (25). ALDEN assessments were performed by multiple independent investigators, minimizing individual bias or scoring errors. In cases where multiple suspected drugs obtained similar ALDEN scores, the causative agent was identified using a hierarchical tie-breaking algorithm. First, temporal plausibility was assessed, with priority given to the drug whose latency from initiation to reaction onset was most consistent with established ALDEN criteria. Second, dechallenge and rechallenge data were considered, giving precedence to drugs associated with symptom improvement upon withdrawal or recurrence upon re-exposure. Third, pharmacological plausibility and prior reports were evaluated, favoring drugs previously documented to cause SJS/TEN or known to carry high risk. Finally, in cases of remaining ambiguity, a consensus review was conducted by all investigators. This multi-step approach ensured objective and reproducible determination of the most probable causative drug in polypharmacy settings while minimizing individual bias.

Following initial screening, the study cohort was narrowed to 150 patients (59.3% females and 40.7% males) aged 18–96 years (median = 50; IQR: 31–63). SJS and TEN were diagnosed in 45.3% and 54.7% of patients, respectively. No additional evaluations beyond those specified in this study protocol were performed.

### Study design

The study was reviewed and approved by the ethics committee of Moscow Clinical Science and Research Center 52 (Protocol No. 12-1223/27.12.2023) and was conducted in accordance with the local legislation and institutional requirements. The EMIAS (The Unified Medical Information and Analytical System of Moscow; EMИAC) automatically records the exact date and time of administration of every medication, including systemic corticosteroids, cyclosporine, IVIG, and other therapies. After collecting demographic details and relevant clinical histories, the documented SCORTEN factors for each patient were obtained from electronic medical records which were assessed either at the time of admission or within 24 h thereafter (14). Patients were untreated until the time of initial SCORTEN assessment. The SCORTEN factors were subsequently evaluated in relation to both length of stay and hospitality survival (Figure 1).

### Statistical analysis

Statistical analyses were performed with Python 3.12 using the packages pandas, scipy, lifelines, matplotlib and seaborn. All continuous variables underwent assumption checks prior to hypothesis testing. The Shapiro–Wilk test demonstrated non-normal distributions for all quantitative variables (all *p* < 0.05), which was consistent with the markedly right-skewed distributions observed on skewness and kurtosis inspection. Homogeneity of variances between outcome groups was evaluated using Levene and Brown–Forsythe tests. Given the non-normality and heterogeneous variances, quantitative variables were summarized as median with interquartile range (Me [IQR]).

Categorical variables were described with absolute (*n*) and relative frequencies (%). Comparison of categorical variables was performed by Pearson's chi-squared test (for expected values >10) or Fisher's exact test (for expected values <10). Odds ratio (OR) with 95% confidential interval (95% CI) were calculated for effect size estimation in univariate analyses. Spearmen's rank correlation (*r*_*s*_) was used to assessment of association between categorical variables.

For survival analyses (time-to-event) was defined as the number of days from admission to in-hospital death. Survivors were censored at discharge. Kaplan–Meier curves were constructed with log-rank tests. Median survival was reported when ≥50% cumulative events were observed. Otherwise, survival was noted as “not reached”.

We used a forced-entry modeling strategy to ensure inclusion of clinically relevant predictors and to avoid unstable variable selection given the limited number of events. The modeling was performed in two stages.

At stage 1, only the seven original SCORTEN factors were entered into the Cox model to evaluate their individual and collective prognostic contribution. At stage 2, comorbidity-related variables (cardiologic, hematologic, rheumatologic, oncological, renal and infectious conditions) were added to the SCORTEN variables.

**Figure 2:**
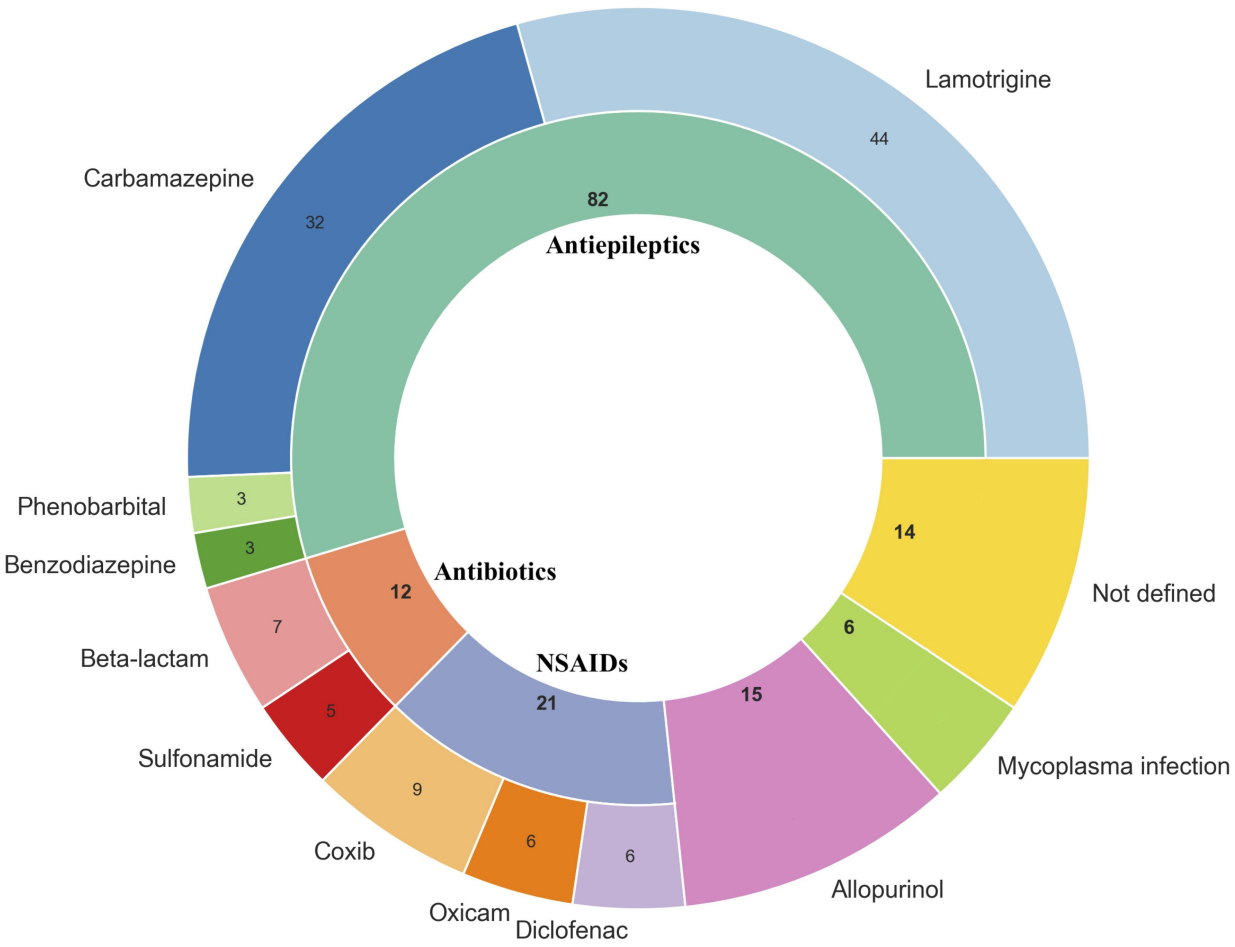
Triggers of SJS/TEN. SJS, Stevens–Johnson syndrome; TEN, toxic epidermal necrolysis; NSAIDs, non-steroidal anti-inflammatory drugs.

**Figure 3:**
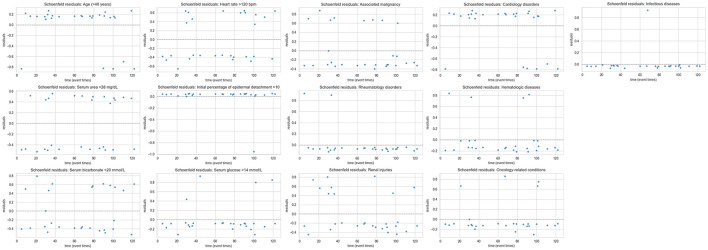
Evaluation of proportional hazards assumptions using Schoenfeld residuals.

Cox proportional hazards models were used to estimate hazard ratios (HR) with 95% CI. Proportional hazards assumptions were evaluated using Schoenfeld residuals (Figure 2) and visual inspection of log–log survival plots for each covariate in both the only SCORTEN (Figure 3) and the SCORTEN and comorbidities (Figure 3) models. No major violations of the proportional hazards assumption were observed.

All tests were two-sided and statistical significance was defined as *p* < 0.05.

## Results

**Figure 4:**
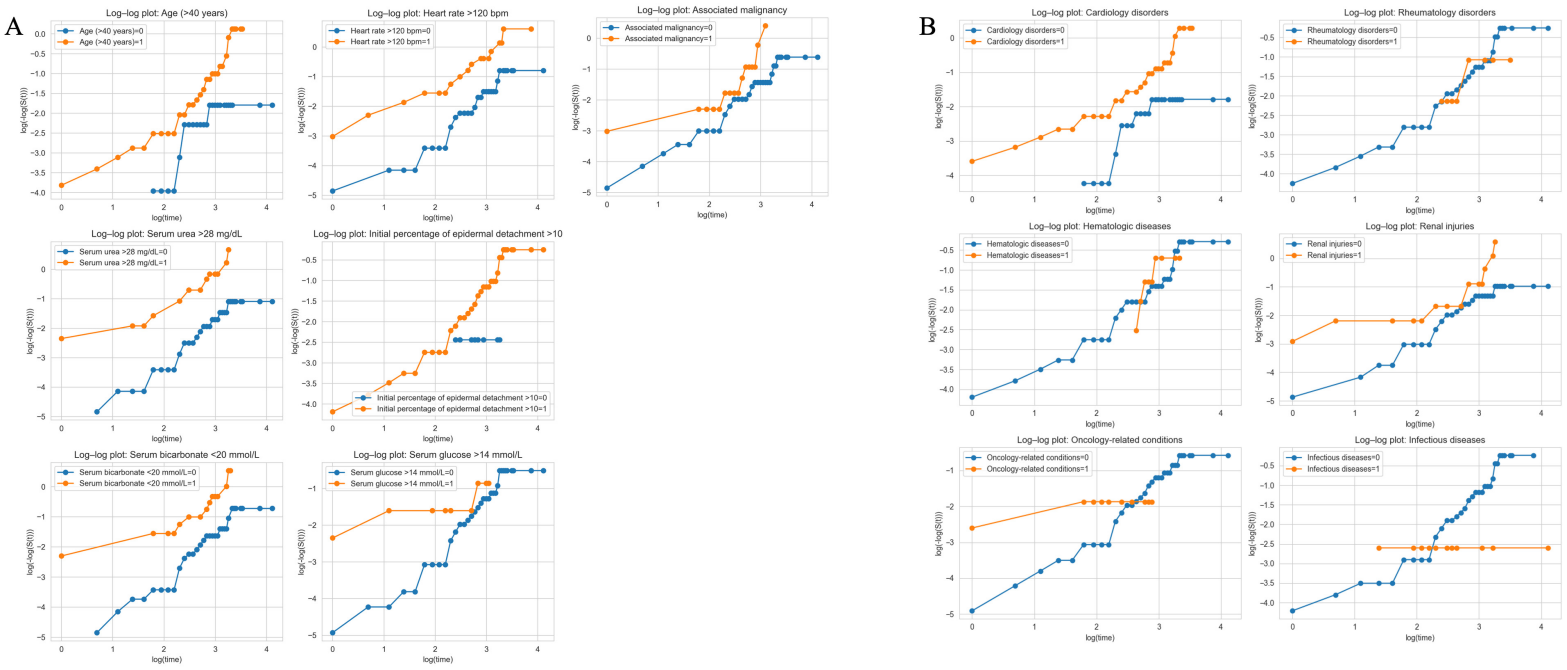
Evaluation of proportional hazards assumptions using visual inspection of log–log survival plots. **(A)** Visual inspection of log–log survival plots for each covariate in the only SCORTEN and the SCORTEN model; **(B)** Visual inspection of log–log survival plots for each covariate in the comorbidities model with SCORTEN factors.

Quantitative variables violated normality assumptions (Shapiro-Wilk test, *p* < 0.05). Suspected triggers of SJS/TEN and patient baseline characteristics are presented in Figure 4 and Table 1 respectively.

**Table 1:** Patient baseline characteristics.

**Characteristics**	**Total (*n* = 150)**
Age (years), Me [IQR]	50 [31–63]
**Gender**, ***n*** **(%)**	
Male	61 (40.7%)
Female	89 (59.3%)
**BMI (kg/m**^2^**)**, ***n*** **(%):**	
<18.5	11 (7.4%)
18–30	98 (65.3%)
>30	41 (27.3%)
**Skin detachment on admission**, ***n*** **(%)**	
SJS	68 (45.3%)
TEN	82 (54.7%)
**Disease duration before SJS/TEN verification (days)**, ***n*** **(%)**	
0–5	89 (59.3%)
>5	61 (40.7%)
Length of Stay (days), Me [IQR]	12 [9–18]
**Hospitalization outcomes**, ***n*** **(%)**	
Favorable	122 (81.3%)
Unfavorable (death)	28 (18.7%)
**Causes of unfavorable outcomes**, ***n*** **(%):**	
Infectious complications	18 (64.3%)
Thromboembolic complications	10 (35.7%)

IQR, interquartile range; Me, median; SJS, Stevens–Johnson syndrome; TEN, Toxic epidermal necrolysis.

### Univariate analysis of SCORTEN factors and comorbidities

We classified SCORTEN factors as significant and non-significant (Table 2). Age above 40 years (OR: 3.53; 95% CI: 1.26–9.9; *p* = 0.01), associated malignancy (OR: 3.35; 95% CI: 1.23–9.09; *p* = 0.03), a heart rate exceeding 120 bpm (OR: 9.42; 95% CI: 3.42–25.64; *p* < 0.001), serum bicarbonate below 20 mmol/L (OR: 7.25; 95% CI: 2.67–19.61; *p* < 0.001) and urea above 28 mg/dL (OR: 14.25; 95% CI: 5.08–40; *p* < 0.001) were significantly associated with mortality. Skin detachment more than 10% and serum glucose had no statistically significant associations (*p* = 0.2; *p* = 0.13, respectively).

**Table 2:** Univariate analysis of SCORTEN factors and comorbidities.

**Characteristics**	**Cohort**	**Outcome**		***P*-value**	**OR; 95% CI**
	**(*****N** =* **150)**	**Favorable (*****N** =* **122; 81.3%)**	**Unfavorable (*****N** =* **28; 18.7%)**		
Age >40, *n* (%)	92 (61.3%)	69 (56.6%)	23 (82.1%)	0.01^a^	3.53; 1.26–9.9
Associated malignancy, *n* (%)	21 (14%)	13 (10.7%)	8 (28.6%)	0.03^b^	3.35; 1.23–9.13
Heart rate >120 bpm, *n* (%)	21 (14%)	9 (7.4%)	12 (42.9%)	<0.001^b^	9.42; 3.43–25.87
Serum urea >28 mg/dL, *n* (%)	22 (14.7%)	8 (6.6%)	14 (50%)	<0.001^b^	14.25; 5.08–39.95
Initial percentage of epidermal detachment >10%, *n* (%)	133 (88.7%)	65 (53.3%)	68 (54.4%)	0.2^b^	4.08; 0.52–32.1
Serum bicarbonate <20 mmol/L, *n* (%)	21 (14%)	10 (8.2%)	11 (39.3%)	<0.001^b^	7.25; 2.68–19.64
Serum glucose >14 mmol/L, *n* (%)	11 (7.3%)	7 (5.7%)	4 (14.3%)	0.13^b^	2.74; 0.74–10.1
**Diagnoses**					
SJS, *n* (%)	68 (45.3%)	62 (50.8)	6 (21.4)		N/A
TEN, *n* (%)	82 (54.7%)	60 (49.2)	22 (78.6)		
**SCORTEN**, ***n*** **(%)**					
0	7 (4.7%)	6 (4.9%)	1 (3.6%)		N/A
1	45 (30%)	44 (36.1%)	1 (3.6%)		
2	47 (31.3%)	46 (37.7%)	1 (3.6%)		
3	27 (17%)	19 (15.6%)	8 (28.6%)		
4	21 (14%)	6 (4.9%)	15 (53.6%)		
5	2 (1.3%)	1 (0.8%)	1 (3.6%)		
6	0 (0%)	0 (0%)	0 (0%)		
7	1 (0.7%)	0 (0.0%)	1 (3.6%)		
**SCORTEN prediction (**≥**4)**, ***n*** **(%)**					
Favorable	126 (84%)	115 (94.3%)	11 (39.3%)		N/A
Unfavorable	24 (16%)	7 (5.7%)	17 (60.7%)		
Comorbidities, *n* (%)	100 (66.7%)	76 (62.3%)	24 (85.7%)	0.03^b^	3.63; 1.19–11.13
**Comorbidities number**, ***n*** **(%)**					
One	60 (40%)	52 (42.6%)	8 (28.6%)	<0.001^a^	N/A
Two	34 (22.7%)	20 (16.4%)	14 (50%)		
Three	6 (4%)	4 (3.3%)	2 (7.1%)		

N/A, not applicable; SJS, Stevens–Johnsons syndrome; SCORTEN, toxic epidermal necrolysis specific-of-illness score; TEN, toxic epidermal necrolysis.

^a^Pearson's chi-square test; ^b^Fisher's exact test.

Comorbid conditions were documented in two-third of the cohort (*n* = 100; 66.7%) with 40% (*n* = 60) having one comorbidity, 22.7% (*n* = 34) having two and 4% (*n* = 6) having three comorbidities. The most frequent comorbidity categories included cardiology disorders (*n* = 74; 49.3%), renal injuries (*n* = 19; 12.7), hematologic diseases (*n* = 16; 10.7%), oncology-related conditions (*n* = 14; 9.3%), infectious diseases (*n* = 14; 9.3%) and rheumatology disorders (*n* = 9; 6%).

Analysis of outcome distribution across comorbidity strata demonstrated a significant, stepwise increase in mortality with higher comorbidity burden (*p* < 0.001). Favorable outcomes were observed in 92% of patients without comorbidities, 86.7% with one comorbidity, but decreased sharply to 58.8% among those with two comorbidities and 66.7% among those with three comorbidities. Correspondingly, mortality increased from 8.0% (without comorbidities) to 13.3% (one), 41.2% (two), and 33.3% (three comorbidities). *Post-hoc* pairwise comparisons revealed that patients with two comorbidities had significantly worse outcomes compared with those without comorbidities (Pearson's chi-square test, *p* = 0.002) and compared with those with one comorbidity (Pearson's chi-square test, *p* = 0.011). No statistically significant differences were identified between the patients without comorbidity vs. one comorbidity or between two vs. three comorbidities (Pearson's chi-square test, *p* > 0.05). These findings align with the observed positive correlation between the number of comorbidities and unfavorable outcome (*r*_*s*_ = 0.29; *p* < 0.001).

### Hospital survival depending on SCORTEN factors

**Figure 5:**
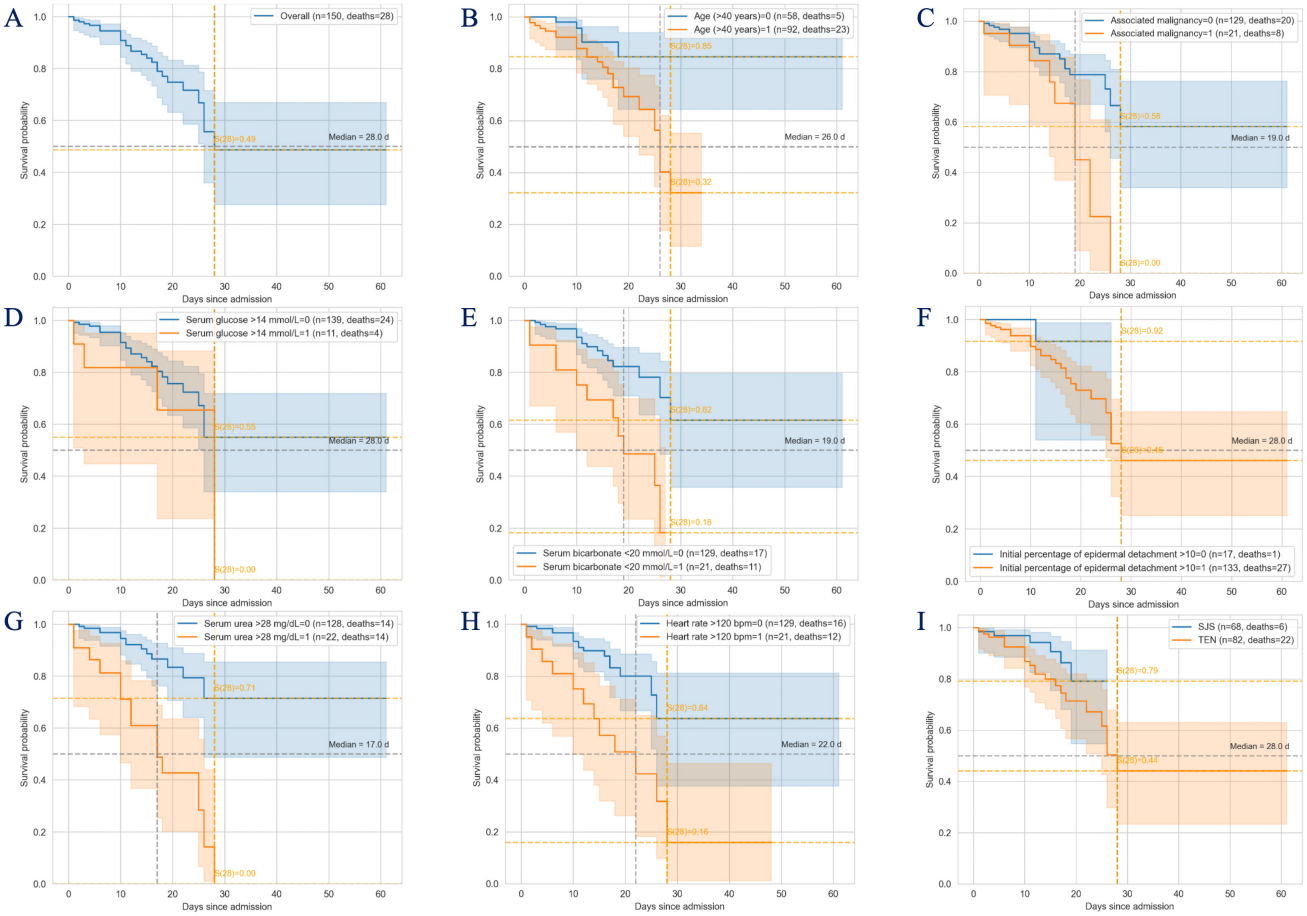
Kaplan-Meier curves of survival probability depending on SCORTEN factors and SJS/TEN diagnosis. **(A)** Hospital survival in SJS/TEN patients; **(B)** age above 40 years; **(C)** associated malignancy and overall; **(D)** serum glucose above 14 mmol/L; **(E)** serum bicarbonate below 20 mmol/L; **(F)** initial percentage of epidermal detachment above 10%; **(G)** serum urea above 28 mg/dL; **(H)** heart rate above 120 bpm; **(I)** SJS/TEN. SJS, Stevens–Johnsons syndrome; TEN, toxic epidermal necrolysis.

In our cohort, the median hospital survival was 28 days (Figure 5A) with an associated mortality risk of 51.3% (95% CI: 33–72.4) on the median survival day. Patients with TEN (*n* = 22; 78.6%) exhibited 3.8 times greater odds of mortality compared to those with SJS (95% CI: 1.44–10; *p* = 0.005). However, Kaplan–Meier survival analysis (Figure 5I) revealed that patients with SJS experienced only a non-significant improvement in hospital survival relative to those with TEN (log-rank *p* = 0.09).

Median hospital survival was determined for patients stratified by SCORTEN factors (Figures 5B–H). Patients with serum urea >28 mg/dL, bicarbonate <20 mmol/L and those with an associated malignancy had markedly reduced median survival time of 17 days (95% CI: 10–26), 19 days (95% CI: 10–N/A), and 19 days (95% CI: 14–26), respectively, with corresponding mortality risks of 57.3% (95% CI: 36.5–79.7), 51.4% (95% CI: 30.2–76.5), and 55% (95% CI: 23.3–91) (Table 3). Although mortality risk estimates varied across the different SCORTEN factors, high-risk variables consistently demonstrated significantly elevated mortality compared to their lower-risk counter variables. Hospital survival differences were statistically significant (log-likehood ratio test, *p* < 0.001).

**Table 3:** Median survival and risk of mortality value.

**Overall/ SCORTEN factors**	**Median survival, days with 95% CI**	**Risk of mortality with 95% CI**
Overall	28 (26—not reached)	51.3 (33–72.4)
**Age (years)**		
>40	26 (22—not reached)	59.8 (38–82.4)
<41	Not reached (<50% events)	15.3 (6.1–35.6)
**Heart rate (bpm)**		
>120	22 (10–28)	57.6 (35.2–81.7)
<121	Not reached (<50% events)	19.9 (11.5–33.3)
**Serum glucose (mmol/L)**		
>14	28 (3–28)	34.5 (11.7–76.4)
<15	Not reached (<50% events)	45 (28.2–66)
**Serum urea (mg/dL)**		
>28	17 (10–26)	57.3 (36.5–79.7)
<29	Not reached (<50% events)	16.6 (9.1–29.3)
**Serum bicarbonate (mmol/L)**		
<20	19 (10—not reached)	51.4 (30.2–76.5)
>19	Not reached (<50% events)	17.7 (10.5–29.1)
**Associated malignancy**		
Presence	19 (14–26)	55 (23.3–91)
Absence	Not reached (<50% events)	21.2 (13.1–33.1)
**Initial percentage of epidermal detachment**		
>10	28 (25—not reached)	53.9 (35.3–74.9)
<11	Not reached (<50% events)	8.3 (1.2–46.1)

SCORTEN, toxic epidermal necrolysis specific-of-illness score; N/A, not applicable.

### Hazards of mortality depending on SCORTEN factors

In the unadjusted Cox proportional-hazards model, several SCORTEN variables demonstrated strong associations with in-hospital mortality (Table 4). The most powerful predictor was serum urea >28 mg/dL, which increased the hazard of death more than fivefold (HR: 5.41; 95% CI: 2.56–11.36; *p* < 0.001). Heart rate >120 bpm was also a robust marker of adverse outcome (HR: 3.61; 95% CI: 1.68–7.69; *p* < 0.001). Metabolic acidosis, reflected by serum bicarbonate <20 mmol/L, significantly elevated mortality risk (HR: 3.65; 95% CI: 1.68–7.87; *p* = 0.001). Additional mortality predictors in the unadjusted model included associated malignancy (HR: 2.93; 95% CI: 1.28–6.76; *p* = 0.01) and age >40 years (HR: 2.97; 95% CI: 1.13–7.81; *p* = 0.03). Serum glucose >14 mmol/L (HR: 2.01; *p* = 0.20) and initial epidermal detachment >10% (HR: 3.24; *p* = 0.25) were not significantly associated with mortality in univariate analysis.

**Table 4:** Hazards of mortality depending on SCORTEN factors.

**SCORTEN factors**	**Unadjusted**		**Adjusted**	
	**HR; 95% CI**	***P*** **-value**	**HR; 95% CI**	***P*** **-value**
Age >40 years	2.97; 1.13–7.81	0.03	2.64; 0.65–10.75	0.18
Associated malignancy	2.93; 1.28–6.76	0.01	3.57; 0.99–12.82	0.05
Serum glucose >14 mmol/L	2.01; 0.69–5.81	0.2	0.69; 0.2–2.36	0.56
Serum bicarbonate <20 mmol/L	3.65; 1.68–7.87	0.001	2.51; 0.92–6.9	0.07
Initial percentage of epidermal detachment >10	3.24; 0.44–23.81	0.25	1.16; 0.14–9.43	0.89
Serum urea >28 mg/dL	5.41; 2.56–11.36	<0.001	4.33; 1.19–15.87	0.03
Heart rate >120 bpm	3.61; 1.68–7.69	<0.001	2.77; 1.02–7.52	0.04

Adjusted—the simple (unadjusted) Cox regression model; bpm, beats per minute; CI, confidential interval; HR, hazard ratio; SCORTEN, toxic epidermal necrolysis specific-of-illness score; Unadjusted—the multivariable Cox proportional hazards regression model.

Three variables retained statistical significance in the adjusted multivariable Cox model. Serum urea >28 mg/dL remained a strong independent predictor (HR: 4.33; 95% CI: 1.19–15.87; *p* = 0.03) confirming its dominant contribution to early mortality risk. Heart rate >120 bpm also preserved significance (HR: 2.77; 95% CI: 1.02–7.52; *p* = 0.04). Associated malignancy demonstrated borderline significance (HR: 3.57; 95% CI: 0.99–12.82; *p* = 0.05). Other SCORTEN items, including age >40 years, serum bicarbonate <20 mmol/L, serum glucose >14 mmol/L, and >10% epidermal detachment, did not retain significance after simultaneous adjustment (all *p* > 0.07).

### Hazards of mortality depending on SCORTEN factors and comorbidities

In unadjusted analyses, several SCORTEN variables demonstrated strong associations with mortality (Table 5). The most pronounced effect was observed for serum urea >28 mg/dL (HR: 5.39; 95% CI: 2.56–11.36; *p* < 0.001). Heart rate >120 bpm (HR: 3.61; 95% CI: 1.69–7.71; *p* < 0.001) and serum bicarbonate <20 mmol/L (HR: 3.64; 95% CI: 1.68–7.90; *p* = 0.001) were also significant predictors. Associated malignancy (HR: 2.93; 95% CI: 1.28–6.75; *p* = 0.011) and age >40 years (HR: 2.97; 95% CI: 1.13–7.82; *p* = 0.028) further increased the risk of death. Among comorbidities, cardiology disorders (HR: 3.58; 95% CI: 1.45–8.85; *p* = 0.006) and renal injuries (HR: 2.68; 95% CI: 1.21–5.95; *p* = 0.016) showed significant univariate associations with mortality. Other comorbidity categories (rheumatologic, hematologic, oncologic, infectious) were not associated with mortality in unadjusted models.

**Table 5:** Hazards of mortality depending on SCORTEN factors and comorbidities.

**SCORTEN factors**	**Unadjusted**		**Adjusted**	
	**HR; 95% CI**	***P*** **-value**	**HR; 95% CI**	***P*** **-value**
Age >40 years	2.97; 1.13–7.82	0.028	1.39; 0.17–11.31	0.76
Heart rate >120 bpm	3.61; 1.69–7.71	<0.001	2.72; 0.95–7.79	0.06
Serum urea >28 mg/dL	5.39; 2.56–11.36	<0.001	5.49; 1.3–23.26	0.021
Initial percentage of epidermal detachment >10	3.23; 0.44–23.83	0.25	1.1; 0.13–9.31	0.93
Serum bicarbonate <20 mmol/L	3.64; 1.68–7.9	0.001	2.32; 0.80–6.70	0.12
Serum glucose >14 mmol/L	2.01; 0.7–5.82	0.2	0.78; 0.19–3.16	0.72
Associated malignancy	2.93; 1.28–6.75	0.011	6.67; 1.25–35.61	0.027
Cardiology disorders	3.58; 1.45–8.85	0.006	2.49; 0.41–15.05	0.32
Rheumatology disorders	0.77; 0.18–3.27	0.73	1.38; 0.28–6.7	0.69
Hematologic diseases	1.02; 0.35–2.95	0.97	0.7; 0.17–2.91	0.62
Oncology-related conditions	1.84; 0.63–5.35	0.26	0.4; 0.11–1.52	0.18
Infectious diseases	0.38; 0.05–2.79	0.34	0.9; 0.11–7.25	0.92
Renal injuries	2.68; 1.21–5.95	0.016	0.55; 0.18–1.7	0.3

Adjusted—the simple (unadjusted) Cox regression model; bpm: beats per minute; CI, confidential interval; HR, hazard ratio; SCORTEN, toxic epidermal necrolysis specific-of-illness score; Unadjusted—the multivariable Cox proportional hazards regression model.

When all SCORTEN factors and comorbidities were entered simultaneously into the multivariable model, only a limited number of predictors remained independently associated with in-hospital mortality. The strongest independent predictor was again serum urea >28 mg/dL, which retained significance with an even higher adjusted effect (HR: 5.49; 95% CI: 1.30–23.26; *p* = 0.021). Associated malignancy remained a significant independent factor with a markedly elevated hazard ratio (HR: 6.67; 95% CI: 1.25–35.61; *p* = 0.027). Heart rate >120 bpm approached statistical significance (HR: 2.72; 95% CI: 0.95–7.79; *p* = 0.06). All remaining SCORTEN parameters including age, serum bicarbonate, serum glucose, and initial epidermal detachment lost statistical significance after adjustment (all *p* > 0.05). Importantly, none of the comorbidity categories aside from malignancy demonstrated independent prognostic value in the adjusted model (all *p* > 0.05).

## Discussion

Drug use was identified as the primary etiological factor in most SJS/TEN cases, although other triggers, such as Mycoplasma spp. infection, were reported in a small subset of patients (*n* = 6; 4%). In our cohort of 150 patients, 86.7% (*n* = 130) of cases were associated with medication exposure, confirming that drugs remain the most common cause of SJS/TEN. The most frequently implicated drug classes were antiepileptic drugs (54.7%), NSAIDs (14%), allopurinol (10%), and antibiotics (8%). These findings are generally in line with previous studies (26–28), although the relative ranking of causative agents differs across reports, likely reflecting variations in study populations, prescribing patterns, and regional drug usage.

SJS and TEN are acute, potentially life-threatening emergencies. In our cohort of 150 patients the overall mortality rate was 18.7% which is higher than some previously reported rates (29, 30) reflecting the know heterogeneity in outcomes across different populations and healthcare setting. Due to the rarity of these conditions estimated at approximately 1.5 cases of TEN and 2 cases of SJS per million population annually the establishment of large patient databases remains challenging (31, 32).

In our study three SCORTEN factors demonstrated statistically significant associations with increased mortality risk. The presence of an associated malignancy was identified as a strong predictor of unfavorable outcome. Our findings regarding malignancy as a prognostic factor in SJS/TEN align partially with previous reports. The SCORTEN system identifies malignancy as a significant risk factor for mortality in SJS/TEN patients (13), a conclusion further supported by Wu et al., who reported higher mortality rates among patients with hepatocellular carcinoma and colorectal cancer (33). Notably, chemotherapy administered within 3 months prior to the onset of SJS/TEN was associated with worse survival outcomes (34). This suggests that systemic cancer treatment, along with the malignancy itself, contributes to poor prognosis. Additionally, malignancy-related complications such as malnutrition and pancytopenia may impair wound healing and further exacerbate clinical outcomes (35).

Elevated serum urea levels were also associated with a significantly increased risk of mortality. Blood urea level is recognized as an independent predictor of mortality in critically ill patients (34). While these levels primarily reflect renal function, they are also influenced by neurohumoral mechanisms involved in fluid homeostasis (36). The pathogenesis of TEN involves dermal infiltration by cytotoxic T lymphocytes (1, 2, 37). This immune-mediated process leads to keratinocyte apoptosis and local inflammation (35), resulting in the formation of subepidermal bullae or widespread epidermal necrolysis without significant dermal involvement (36). The extensive destruction of the epidermal barrier causes substantial fluid loss, leading to hypovolemic and distributive shock (a pathophysiological state analogous to burn shock) (37). In severe burn injuries involving more than 20% of the total BSA, early non-inflammatory acute kidney injury is a well-documented complication (38, 39). In the context of TEN, however, early renal impairment may result not only from hypovolemia but also from the systemic effects of circulating pro-inflammatory cytokines and complement activation (40, 41). Notably, the presence of renal failure requiring renal replacement therapy prior to the onset of TEN symptoms has been identified as the strongest predictor of mortality in the ABCD-10 prognostic score (19). Furthermore, renal dysfunction at the time of admission has been shown to correlate with a poorer clinical outcome.

Tachycardia, defined as a heart rate exceeding 120 bpm, was another independent predictor of mortality, consistent with previous findings that link elevated heart rate to systemic inflammatory response and cardiovascular stress in patients with TEN (42).

Other SCORTEN variables, such as age, percentage of body surface area involved, serum bicarbonate levels and serum glucose levels did not reach statistical significance in the Moscow cohort of patients.

A common limitation is the subjective nature of BSA, which can vary among clinicians depending on whether they assess only areas of desquamation or include skin with bullae. These differences may be attributable to the relatively small sample size or demographic variations compared to those in the original SCORTEN validation studies. Similarly, Zavala et al showed that BSA was not a statistically significant predictor (43).

Age is a well-recognized risk factor for mortality in SJS/TEN. In the original SCORTEN study, the mean age of participants was 42.3 years, and an age threshold of over 40 years was identified as a significant predictor of mortality (13). Similarly, the ABCD-10 score used a cut-off age of 50 years as a mortality risk indicator (19). However, findings across studies have not been entirely consistent. In our cohort, age was significantly associated with mortality in univariate analysis, whereas Thakur et al. (44) reported no significant association between age and survival in patients with SJS/TEN or TEN. These discrepancies highlight population-specific variability in the prognostic impact of age, which may reflect differences in demographics, comorbidities, or healthcare practices.

The reliability of certain laboratory parameters included in the SCORTEN score has been questioned in the literature (45–47). In our study some critically ill patients received parenteral nutrition and frequently required insulin therapy. In these conditions, serum glucose levels measured during the first 5 days of observation do not reliably reflect disease severity or predict prognosis. Consistent with this, our analysis did not identify serum glycemia as a significant predictor of mortality in our patient cohort as has been shown in previously published studies (45).

Low serum bicarbonate levels have been identified as a valuable prognostic marker in critically ill patients, particularly for predicting early mortality and the risk of acute kidney injury (48). In the context of TEN, low bicarbonate concentration has been reported as one of the most significant predictors of poor outcomes (49). However, no association was demonstrated between reduced serum bicarbonate levels and increased mortality in our cohort. This may be attributed to advancements in the therapeutic strategies employed in patient management.

Despite its good performance, the SCORTEN scale has problems with both accuracy and predictive power. Numerous studies have questioned SCORTEN's predictive ability and emphasized the necessity to reevaluate the current parameters to create a Re-SCORTEN (50, 51). Another mortality-prediction model, ABCD-10, has been developed to reliably predict in-hospital mortality from SJS/TEN because SCORTEN has been shown to overestimate and underestimate SJS/TEN-related in-hospital mortality in a variety of populations (19).

Overall, a number of SCORTEN factors including malignancy, higher blood urea nitrogen, and heart rate >120 bpm were associated with increased mortality (52). However, in our investigation, there was no significant correlation between unfavorable outcomes and other SCORTEN components, such as age, percentage of body surface area involved, serum bicarbonate levels and serum glucose. Since the accurate risk stratification plays a key role in enhancing patient assessment, guiding management decisions, and more precisely estimating the survival benefits of immunomodulatory therapy, it is crucial to refine SCORTEN factors or to develop a more appropriate score system.

Given the rarity, heterogeneity, and high mortality associated with SJS/TEN, our findings underscore the need for further research aimed at improving risk stratification and early clinical assessment. Although our study identified malignancy, elevated serum urea, and tachycardia as significant predictors of mortality, other established SCORTEN variables did not reach statistical significance, reflecting both the limitations of our sample size and the challenges inherent to studying such an uncommon disorder. Future multicenter prospective studies with larger and more diverse patient populations are essential to validate these observations, refine existing prognostic tools such as SCORTEN, and determine whether additional variables—such as systemic inflammatory markers, early hemodynamic parameters, or measures of organ dysfunction—improve predictive accuracy. Moreover, exploring the biological mechanisms linking systemic inflammation, fluid loss, renal impairment, and cardiovascular instability may offer valuable insights into disease pathophysiology and support the development of more targeted therapeutic strategies. Such efforts may ultimately contribute to more personalized management approaches and better clinical outcomes in patients with SJS/TEN.

## Limitations

This study has several inherent limitations. First, its retrospective, single-center design may limit the generalizability of the findings to other populations or healthcare settings. Second, although all data were extracted from electronic medical records and independently verified by multiple reviewers, the reliance on retrospective documentation introduces the potential for incomplete, missing, or inconsistent information. Third, the relatively small sample size, a consequence of the rarity of SJS/TEN, may restrict the statistical power to detect subtle associations. Fourth, while the timing and administration of systemic therapies were confirmed using the EMIAC system, any interventions occurring outside this system could not be captured. Fifth, although SCORTEN assessments were independently performed by several investigators to minimize bias, some degree of subjective judgment is unavoidable in retrospective evaluations. Additionally, the study did not adjust the analyses for treatment regimens, and therefore the potential influence of administered therapies on the relationship between SCORTEN items and mortality cannot be fully excluded. Despite these limitations, the study provides valuable real-world evidence regarding SCORTEN performance and highlights areas where further prospective research is needed to support validation and refinement of prognostic scoring systems.

## Data Availability

The raw data supporting the conclusions of this article will be made available by the authors, without undue reservation.
